# Thermal biology and swimming performance of Atlantic cod (*Gadus morhua*) and haddock (*Melanogrammus aeglefinus*)

**DOI:** 10.7717/peerj.7784

**Published:** 2019-10-01

**Authors:** Tommy Norin, Paula Canada, Jason A. Bailey, A. Kurt Gamperl

**Affiliations:** 1 Department of Ocean Sciences, Memorial University of Newfoundland, St. John’s, NL, Canada; 2 DTU Aqua: National Institute of Aquatic Resources, Technical University of Denmark, Kgs. Lyngby, Denmark; 3 Oceanic Observatory of Madeira, Agência Regional para o Desenvolvimento da Investigação Tecnologia e Inovação, Funchal, Portugal; 4 CIIMAR—Centro Interdisciplinar de Investigação Marinha e Ambiental, Universidade do Porto, Matosinhos, Portugal; 5 Vattenbrukscentrum Ost, East Region Aquaculture Centre, Vreta Kloster, Sweden

**Keywords:** Critical swimming speed, Metabolic rate, Critical thermal maximum, Physiology, Fish, Climate change, Thermal tolerance, Temperature, Climate warming

## Abstract

Atlantic cod (*Gadus morhua*) and haddock (*Melanogrammus aeglefinus*) are two commercially important marine fishes impacted by both overfishing and climate change. Increasing ocean temperatures are affecting the physiology of these species and causing changes in distribution, growth, and maturity. While the physiology of cod has been well investigated, that of haddock has received very little attention. Here, we measured the metabolic response to increasing temperatures, as well as the critical thermal maximum (CT_max_), of cod acclimated to 8 and 12 °C and haddock acclimated to 12 °C. We also compared the swimming performance (critical swimming speed, *U*_crit_) of cod and haddock at 12 °C, as well as the *U*_crit_ of 12 °C-acclimated cod acutely exposed to a higher-than-optimal temperature (16 °C). The CT_max_ for cod was 21.4 and 23.0 °C for 8- and 12 °C-acclimated fish, respectively, whereas that for the 12 °C-acclimated haddock was 23.9 °C. These values were all significantly different and show that haddock are more tolerant of high temperatures. The aerobic maximum metabolic rate (MMR) of swimming cod remained high at 16 °C, suggesting that maximum oxygen transport capacity was not limited at a temperature above optimal in this species. However, signs of impaired swimming (struggling) were becoming evident at 16 °C. Haddock were found to reach a higher *U*_crit_ than cod at 12 °C (3.02 vs. 2.62 body lengths s^−1^, respectively), and at a lower MMR. Taken together, these results suggest that haddock perform better than cod in warmer conditions, and that haddock are the superior swimmer amongst the two species.

## Introduction

Overfishing and climate change, independently or in combination, are affecting fish populations in the world’s oceans (Perry et al., 2005; Cheung et al., 2009, 2013; Pecl et al., 2017). Poleward migrations and distribution shifts of fishes have been observed in response to warming for various cold-water species (Perry et al., 2005; Cheung, Watson & Pauly, 2013; Pörtner et al., 2014; Fossheim et al., 2015) and increased temperatures are affecting their physiology (Pörtner & Knust, 2007; Hofmann & Todgham, 2010; Pörtner & Peck, 2010; Audzinjoyte et al., 2019) resulting in, amongst other things, reduced size-at-age and lower fishery yields (Cheung, Watson & Pauly, 2013; Daufresne, Lengfellner & Sommer, 2009; Baudron, Needle & Marshall, 2011).

In addition to the relatively slow, but steady, effect of human-induced climate warming (Bradshaw & Holzapfel, 2006; Lima & Wethey, 2012), the temperature experienced by individual fish varies significantly both within and across populations. This is due to differences or variations in physiology and behaviour (Kobler et al., 2009; Killen, 2014), geographic location, year-to-year and seasonal temperatures, depth, current, and/or age of the fish (Malmberg & Blindheim, 1994; Ottersen, Michaelsen & Nakken, 1998; Morita et al., 2010; Rijn et al., 2017). Thus, fish are faced with both temporal and spatial fluctuations in ambient temperature, which requires adjustments in physiological and behavioural traits over both short and long time scales. Since key physiological functions such as growth, metabolic rate, and swimming performance have species-specific temperature optima that combine to determine a species’ preferred temperature (Pörtner & Peck, 2010; Fry, 1971; Clark, Sandblom & Jutfelt, 2013; Speers-Roesch & Norin, 2016), the capacity for physiological adaptation and acclimation will, along with behavioural adaptations, define the temperature window for growth and reproduction as required for successful long-term maintenance of a population (Pörtner, 2002; Wang & Overgaard, 2007; Holt & Jørgensen, 2015). On shorter time-scales, the thermal dependence of swimming performance and metabolic rate is important when fish are migrating and foraging through thermally variable environments (Neat & Righton, 2007; Neuenfeldt, Andersen & Hinrichsen, 2009) or actively tracking a preferred temperature (Ottersen, Michaelsen & Nakken, 1998; Claireaux et al., 1995; Castonguay et al., 1999; Begg & Martinesdottir, 2002; Cavole et al., 2016).

Aerobic scope (AS), the difference between the aerobic maximum metabolic rate (MMR) and the standard (resting) metabolic rate (SMR), is a measure of the energy available for physical activity, growth, and reproduction (Fry, 1971; Clark, Sandblom & Jutfelt, 2013; Farrell, 2016). It has been suggested that AS determines both the thermal preference and thermal tolerance of fishes (Pörtner & Knust, 2007; Pörtner & Farrell, 2008), although this is currently being debated (Clark, Sandblom & Jutfelt, 2013; Holt & Jørgensen, 2015; Gräns et al., 2014; Norin, Malte & Clark, 2014; Schulte, 2015; Pörtner, Bock & Mark, 2017, 2018; Jutfelt et al., 2018). Nonetheless, measurements of swimming performance and metabolic rate provide insights into the thermal biology of a species. Generally, swimming performance and metabolic rate increase with rising water temperature up to an optimum (Brett, 1971; Gamperl et al., 2002), after which performance is decreased (Lefevre, 2016). However, there are also examples where the thermal optimum can approach the species’ upper critical (or lethal) temperature (Clark, Sandblom & Jutfelt, 2013; Gräns et al., 2014; Norin, Malte & Clark, 2014; Lefevre, 2016).

Atlantic cod (*Gadus morhus*) and haddock (*Melanogrammus aeglefinus*) are two very important commercial species affected by both overfishing (Beaugrand et al., 2003; Brander, 2005; Cardinale et al., 2012) and ongoing climatic warming (Perry et al., 2005; Cheung et al., 2013; Cheung, Watson & Pauly, 2013; Baudron, Needle & Marshall, 2011; Rogers et al., 2011). Furthermore, the two species overlap broadly in their distribution and ecology (Eriksen et al., 2012; Renaud et al., 2012), with the haddock appearing to prefer slightly warmer temperatures than cod (Cheung, Watson & Pauly, 2013; Eriksen et al., 2012) and responding differently to temperature in terms of distribution, growth, and age-at-maturity (Baudron, Needle & Marshall, 2011; Eriksen et al., 2012). Despite the similarities (and dissimilarities) between the two species, little effort has been made to directly compare the physiological performance of cod and haddock under different temperature scenarios; information that is important for understanding how these species may respond to climate warming, and how they may compete both amongst each other and with other species they may encounter as their distributions continue to change (Renaud et al., 2012).

The effect of temperature on the swimming performance and metabolic rate of cod has been investigated in several studies (Claireaux et al., 1995; Schurmann & Steffensen, 1997; Claireaux et al., 2000; Sylvestre et al., 2007; Tirsgaard, Behrens & Steffensen, 2015). Surprisingly, however, the effect of acclimation temperature on these parameters and on upper thermal tolerance (upper critical temperature) of this species has not been extensively investigated (but see Tirsgaard, Behrens & Steffensen, 2015; Kelly et al., 2014). The metabolic physiology of haddock has not received nearly as much attention (Peck, Buckley & Bengtson, 2005; Lankin et al., 2008; Perez-Casanova, Lall & Gamperl, 2010; Tytler, 1969) and, with the exception of Tytler (1969), the few studies that exist on haddock have focused on routine metabolic rate (RMR) (Peck, Buckley & Bengtson, 2005; Lankin et al., 2008; Perez-Casanova, Lall & Gamperl, 2010), with swimming performance and active or maximum metabolic rates being largely overlooked. Clearly, such information would be beneficial, as maximum metabolic performance and swimming speed are important not only for these species’ migration and foraging, but likely also for their susceptibility to capture by (or ability to escape from) fishing gear (Pörtner & Peck, 2010; He, 1993; Breen et al., 2004; Hollins et al., 2018; Thambithurai et al., 2018).

Based on the above, the objectives of the present study were to: (1) examine the influence of acclimation temperature on the upper thermal tolerance (critical thermal maximum, CT_max_) of cod by exposing fish from two acclimation groups (8 and 12 °C) to an acute (2 °C h^−1^) increase in water temperature until loss of equilibrium; (2) determine the effect of an acute temperature increase up to a supra-optimal temperature (i.e. from 12 to 16 °C) on the swimming performance and metabolic rate of cod using the well-established critical swimming speed (*U*_crit_) test (cf., Brett, 1964; Farrell, 2007); and (3) compare the thermal tolerance, swimming performance, and metabolic rates of cod and haddock acclimated to 12 °C (including examining whether metabolic parameters (e.g. MMR and AS) in these species differ when obtained using CT_max_ vs. *U*_crit_ tests).

## Materials and Methods

These studies were conducted in accordance with the guidelines of the Canadian Council on Animal Care and approved by the Institutional Animal Care Committee of Memorial University of Newfoundland (MUN), Canada (protocol #05-01-KG).

### Fish

The cod used in these experiments were spawned from broodstock collected in Placentia Bay (Newfoundland, Canada), and reared at the Dr. Joe Brown Aquatic Research Building (JBARB) at MUN’s Ocean Sciences Centre (OSC). The haddock were spawned and reared at the National Research Council’s Sandy Cove aquaculture facility (Nova Scotia, Canada) until a size of 5–10 g, and then shipped to the OSC. Both species were held indoors at the JBARB in 3,000 L circular fibreglass tanks containing oxygenated seawater (~32 ppt salinity) at 10 °C. At least 3 weeks before experiments began, water temperature was changed to the experimental acclimation temperatures (8 ± 1 or 12 ± 1 °C). Fish used in the swimming performance experiments were exposed during this period to a current of approximately 1 body length per second (BL s^−1^). This current was created in the tanks using a vertical spray bar that received water from the tank inflow line (for the cod) or a submersible pump (Little Giant Co., Oklahoma City, OK, USA) placed at the bottom of the tank (for the haddock). During the holding and acclimation periods, the fish were exposed to an ambient photoperiod and fed commercial pellets, twice a day, to satiation. However, feeding was suspended 24 h before experimentation. All experiments were performed from August to November 2004.

### Experimental details (respirometry)

Measurements of oxygen uptake rate ${\rm (}{\dot M_{{{\rm O}_2}\hskip-1pt}})$, obtained using intermittent-closed respirometry (Clark, Sandblom & Jutfelt, 2013; Steffensen, 1989), were used as proxies for the fish’s aerobic metabolic rates at the different swimming speeds and temperatures (detailed below).

All experiments were conducted using a modified Blažka swim tunnel respirometer (6.81 L volume), composed of two Plexiglas tubes (10 and 15 cm in diameter, one inside the other), with conical-shaped end caps, an impellor, and a honeycomb plastic grid at the front to promote a laminar water flow (Waterloo Biotelemetry Institute, University of Waterloo, Waterloo, ON, Canada). In this swim tunnel, the fish swam in a stationary position in the swimming section (37 cm in length) of the inside tube, and water speed (i.e. revolutions of the impellor) was controlled by an electric motor. Water was continuously supplied to the swim tunnel at a rate of 5 L min^−1^ by a submersible pump (Little Giant Pump Co., Oklahoma City, OK, USA) placed in a large (120 L) reservoir adjacent to the swim tunnel. The water temperature in the reservoir was controlled using thermostatically-controlled circulating water baths (model 1013S; Fisher Scientific, Pittsburgh, PA, USA), which pumped water through stainless steel coils immersed in the reservoir. Oxygen tension in the reservoir was maintained at air saturation by bubbling air and/or pulsing pure oxygen into the water as needed. Water temperature and oxygen concentration in the swim tunnel were continuously monitored during the experiments using a peristaltic pump (Masterflex L/S model 77200-12; Cole-Palmer, Vernon Hills, IL, USA) to draw water past a galvanic oxygen electrode with thermal sensor (CellOx 325; WTW, Weilheim, Germany). This sensor was housed in a flow-through chamber (D-201; WTW, Weilheim, Germany) in an external circuit comprised of tubing with very low gas permeability (Tygon Food^®^; Cole Palmer, Inc., Vernon Hills, IL, USA). The oxygen electrode was connected to an oxygen metre (Oxi 342; WTW, Weilheim, Germany) with automatic temperature compensation, and water oxygen concentration was recorded in mg O_2_ L^−1^. The front portion of the respirometer was covered with black plastic during experiments to provide a darkened refuge and to minimise disturbance of the fish. This darkened section encouraged the fish to maintain its swimming position towards the front of the swim tunnel. The rear of the swimming section contained a stainless steel grid which, in order to prevent the fish from resting on the grid during swimming experiments, was connected to electrodes so that mild electrical stimuli (<5 V, ~0.2 A) could be applied.

${\dot M_{{{\rm O}_2}}}$ measurements were made by stopping the flow of water into the swim tunnel respirometer and recording the drop in water oxygen content caused by the fish respiring inside the respirometer, after which ${\dot M_{{{\rm O}_2}}}$ (in mg O_2_ h^−1^ kg^−1^) was calculated as:

$${\dot M_{{{\rm O}_2}}} = V \cdot {\alpha } \cdot {\rm }{M^{ - 1}}$$

where, *V* is volume of the respirometer and external circuit (6.81 L) minus the volume of the fish (assuming a density of 1 kg L^−1^), α is the decline in oxygen concentration during the closed phase of the respirometry cycle (mg O_2_ L^−1^ h^−1^), and *M* is body mass of the fish (kg). ${\dot M_{{{\rm O}_2}}}$ measurements were allometrically scaled (to a standard body mass of 100 g) using a mass-scaling exponent of 0.80 (Saunders, 1963; Reidy et al., 1995) as:

$${\dot M_{{{\rm O}_{2,{\rm scaled}}}}}\; = \; {\dot M_{{{\rm O}_{2,{\rm measured}}}}} \cdot {\rm }{({M_{{\rm measured}}} \cdot {M_{{\rm scaled}}}^{ - 1})^{(1 - b)}}$$

where ${\dot M_{{{\rm O}_{2,{\rm scaled}}}}}$ is the standardised (body-mass-adjusted) ${\dot M_{{{\rm O}_2}}}$ value, ${\dot M_{{{\rm O}_{2,{\rm measured}}}}}$ is the measured ${\dot M_{{{\rm O}_2}}}$ value, *M*_measured_ is the measured body mass, *M*_scaled_ is the body mass to which the ${\dot M_{{{\rm O}_2}}}$ values were standardised (100 g), and *b* is the mass-scaling exponent (0.80).

### Experimental protocols

#### Metabolic rate and critical thermal maximum

Individual fish were transferred from the holding tank to the swim tunnel respirometer the evening before each trial and allowed to recover for at least 15 h. During this time, water was continuously supplied from the reservoir via the submersible pump. Oxygen levels were maintained at >90% air saturation, water velocity was 7.5 cm s^−1^ (~0.4 BL s^−1^) to ensure mixing within the swim tunnel, and water temperature was maintained at the fish’s acclimation temperature (8 or 12 °C). Thermal challenges began at 08:00–10:00 the next morning and lasted 6–8 h. In these experiments, the water temperature was increased at a rate of 2 °C h^−1^ until the fish lost equilibrium. The temperature at loss of equilibrium was recorded as the fish’s CT_max_. ${\dot M_{{{\rm O}_2}}}$ was measured over 12 min intervals, starting at 8 or 12 °C and then for every 1 °C increase in temperature until CT_max_. Immediately after the fish reached CT_max_, they were removed from the swim tunnel and anaesthetised in 0.1 g L^−1^ MS-222. Fork length (L, cm) and body mass (M, g) were measured and the condition factor (K) was calculated as K = 100 ∙ M ∙ L^−3^.

The ${\dot M_{{{\rm O}_2}}}$ at 8 °C (cod) or 12 °C (cod and haddock) was taken as the fish’s RMR, and the maximum ${\dot M_{{{\rm O}_2}}}$ recorded during the thermal challenges was taken as the fish’s temperature-induced maximum metabolic rate (MMR_*T*_). In the absence of SMR measurements, temperature-induced aerobic scope (AS_*T*_) was approximated as MMR_*T*_ – RMR, bearing in mind that AS is defined as MMR – SMR.

Acute thermal sensitivity (i.e. temperature coefficient, *Q*_10_) of metabolic rate was calculated as:

$${Q_{10}} = {\left( {\displaystyle{{{{\dot M}_{{{\rm O}_{2,2}}}}} \over {{{\dot M}_{{{\rm O}_{2,1}}}}}}} \right)^{\left( {\textstyle{{10} \over {{T_2} - {T_1}}}} \right)}}$$

where ${\dot M_{{{\rm O}_{2,2}}}}$ is the ${\dot M_{{{\rm O}_2}}}$ at the higher temperature (*T*_2_) and ${\dot M_{{{\rm O}_{2,1}}}}$ is the ${\dot M_{{{\rm O}_2}}}$ at the lower temperature (*T*_1_) between which *Q*_10_ is calculated.

#### Metabolic rate and swimming performance

As per the protocol for the CT_max_ tests, fish were transferred into the swim tunnel the evening prior to the start of each trial, and allowed to settle in the tunnel at a low water velocity of 7.5 cm s^−1^ for at least 15 h. Two different protocols were used in the swimming performance (*U*_crit_) experiments: First, to examine whether the swimming performance and activity metabolism of 12 °C-acclimated cod and haddock differed, ${\dot M_{{{\rm O}_2}}}$ was recorded for both species at 12 °C, then water velocity was increased in increments of 5 cm s^−1^ (starting from 7.5 cm s^−1^) every 20 min until the fish fatigued. At each swimming speed, ${\dot M_{{{\rm O}_2}}}$ was measured over 12 min, beginning 5 min after the speed was increased. Second, to examine the effect of an acute temperature increase on the swimming and metabolic performance of cod, another group of 12 °C-acclimated cod were exposed to a 2 °C h^−1^ increase in water temperature to 16 °C, held at 16 °C for a further 2 h, and then subjected to a *U*_crit_ test as described above.

Exhaustion in all experiments was determined as the inability of the fish to separate itself from the rear grid of the respirometer after two or three mild electrical stimuli. At the end of the *U*_crit_ test, the fish were removed from the swim tunnel and anaesthetised in 0.1 g L^−1^ MS-222 for measurements of body mass and fork length. Condition factor was calculated as described above.

The ${\dot M_{{{\rm O}_2}}}$ of fish swimming at 7.5 cm s^−1^ at 12 °C (cod and haddock) or 16 °C (cod) was taken as the fish’s RMR. The maximum ${\dot M_{{{\rm O}_2}}}$ recorded (usually at, or immediately before, the maximum swimming speed) was taken as the swimming-induced MMR (MMR_*S*_). SMR was estimated by extrapolating the ${\dot M_{{{\rm O}_2}}}$ vs. swimming speed relationship back to a swimming speed of zero. For the purpose of comparing with AS_*T*_, swimming-induced aerobic scope (AS_*S*_) was also approximated as MMR_*S*_ – RMR, which underestimated the ‘true’ AS (i.e. MMR – SMR) by ~20% (see Results for details).

Critical swimming speed was calculated as:

$${U_{{\rm crit}}} = U + {\rm }({t_f} \cdot {U_i} \cdot {t_i}^{ - 1})$$

where *U* is the velocity at which the fish swam for the entire time increment, *U_i_* is the velocity increment (5 cm s^−1^), *t_f_* is the time elapsed from the last change in water velocity to fatigue, and *t_i_* is the time increment between stepwise increases in water velocity (20 min).

The fish’s cost of transport (COT, mg O_2_ km^−1^ kg^−1^) was calculated as either their gross COT (COT_gross_) by dividing their absolute ${\dot M_{{{\rm O}_2}}}$ at a given velocity by swimming speed, or as their net COT (COT_net_) by subtracting SMR from their ${\dot M_{{{\rm O}_2}}}$ before dividing by swimming speed. The optimal swimming speed (*U*_opt_) was then calculated by fitting third-order polynomials to the COT_net_ vs. *U* relationship for each fish, and finding the swimming speed (in 0.1 cm s^−1^ bins) corresponding to the minimum COT_net_ (i.e. COT_min_).

### Statistics

Statistical analyses were performed using SigmaPlot 11 (Systat Software Inc., San Jose, CA, USA). One-way ANOVAs, followed by Holm–Sidak post hoc tests, were performed to assess differences between groups with respect to body mass, fork length, condition factor, SMR, RMR, maximum metabolic rate (MMR_*T*_ or MMR_*S*_), aerobic scope (AS_*T*_ or AS_*S*_), CT_max_, *U*_crit_, *U*_opt_, and COT_min_. All data presented in the text, figures, and tables are means ± s.e.m. The level of statistical significance for all tests was *P* < 0.05, but differences at the *P* < 0.10 level are also noted in the tables.

## Results

### Metabolic rate and critical thermal maximum

**Figure 1:**
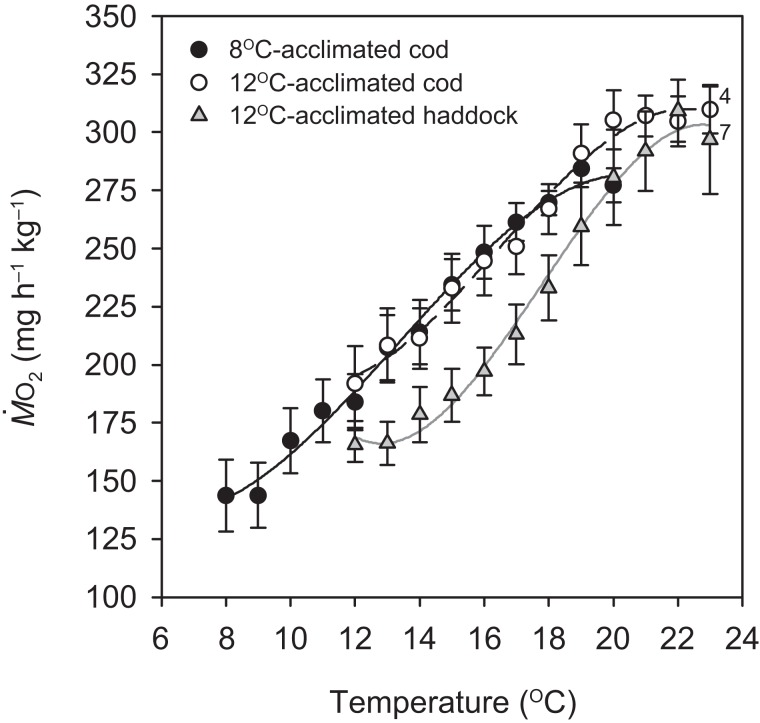
Metabolic rate of cod and haddock during warming. Metabolic rate (${\rm }{\dot M_{{{\rm O}_2}}}$; means ± s.e.m.) for cod acclimated to 8 °C (black circles, solid black line; *n* = 9), cod acclimated to 12 °C (white circles, broken black line; *n* = 9), and haddock acclimated to 12 °C (grey triangles, solid grey line; *n* = 8) as measured during 2 °C h^−1^ warming challenges (i.e. critical thermal maximum, CT_max_, tests). Lines are third order polynomials. Numbers next to symbols indicate reduced sample size due to variability in the CT_max_ measurement or exclusion of individuals exhibiting abnormally high activity levels (i.e. struggling).

Routine metabolic rate for the 8 °C-acclimated cod was significantly lower than the RMR for 12 °C-acclimated cod by 25.2% (*P* = 0.046; Table 1), but not different from the RMR of 12 °C-acclimated haddock (*P* = 0.240). There was also no difference in RMR between the species at 12 °C (*P* = 0.174) (Table 1). Except for the first 1 °C increase for the 8 °C-acclimated cod and 12 °C-acclimated haddock, oxygen uptake $({\dot M_{{{\rm O}_2}\hskip-1pt}})$ increased at a relatively constant rate in all groups until MMR_*T*_ was reached at 18.9 ± 0.4, 20.3 ± 0.7, and 21.7 ± 0.5 °C for 8 °C-acclimated cod, 12 °C-acclimated cod, and 12 °C-acclimated haddock, respectively (Fig. 1). The *Q*_10_ value for ${\dot M_{{{\rm O}_2}}}$ from 8 to 19 °C (i.e. from RMR to MMR_*T*_) for 8 °C-acclimated cod was 2.14 ± 0.24, whereas it was 1.97 ± 0.27 from 12 to 20 °C for 12 °C-acclimated cod. These values are slightly higher than the *Q*_10_ value from 12 to 22 °C for 12 °C-acclimated haddock (1.87 ± 0.04), but no significant differences were detected in *Q*_10_ values between groups (*P* > 0.05). Close to the fish’s CT_max_, ${\dot M_{{{\rm O}_2}}}$ for all three groups tended to plateau or decrease slightly (Fig. 1). This plateau just before CT_max_ was often coincident with struggling, an observation suggesting that the fish were under physiological and/or behavioural stress.

**Table 1:** Metabolic rate and thermal tolerance of cod and haddock. Routine metabolic rate (RMR) measured at the acclimation temperature as well as temperature-induced maximum metabolic rate (MMR*_T_*), temperature-induced aerobic scope (AS*_T_*), and critical thermal maximum (CT_max_) for cod acclimated to 8 and 12 °C and haddock acclimated to 12 °C, as measured during the 2 °C h^−1^ warming challenges (i.e. CT_max_ tests). Metabolic rates scaled to a body mass of 100 g (using a metabolic mass-scaling coefficient of 0.80) are presented in italics in parentheses. Comparisons between groups were made using one-way ANOVAs followed by Holm–Sidak post hoc tests.

	8 °C-acclimated cod	12 °C-acclimated cod	12 °C-acclimated haddock
*n*	9	9	8
M (g)	73.0 ± 1.7^a^	64.6 ± 3.6^b^	64.4 ± 1.56^b^
L (cm)	21.6 ± 0.2^a^	20.8 ± 0.3^b^	18.7 ± 0.2^c^
K	0.72 ± 0.02^a^	0.71 ± 0.03^a^	0.99 ± 0.03^b^
RMR (mg O_2_ h^−1^ kg^−1^)	143.6 ± 15.5^a^ (*134.7 ± 14.4*^*(d)*^)	191.9 ± 16.2^b^ (*175.3 ± 14.6*^*(e)*^)	165.5 ± 7.0^a,b^ (*151.4 ± 6.5*^*(d,e)*^)
MMR_*T*_ (mg O_2_ h^−1^ kg^−1^)	285.5 ± 7.9^a^ (*267.9 ± 7.1*^*a*^)	315.2 ± 8.4^b^ (*290.8 ± 6.9*^*b*^)	331.0 ± 6.3^b^ (*302.8 ± 4.8*^*b*^)
AS_*T*_ (mg O_2_ h^−1^ kg^−1^)	141.8 ± 18.3^a,b^ (*133.2 ± 17.1*^*(d,e)*^)	123.3 ± 16.4^a^ (*115.6 ± 16.4*^*(d)*^)	168.0 ± 6.1^b^ (*151.4 ± 5.6*^*(e)*^)
CT_max_ (°C)	21.4 ± 0.3^a^	23.0 ± 0.3^b^	23.9 ± 0.3^c^

**Notes:**

a,b,c Significant differences between groups at *P* < 0.05

d,e,f Differences at P < 0.10 (pairwise comparisons) Values are means ± s.e.m.

*n*, number of experimental animals; M, body mass; L, fork length; K, condition factor.

Temperature-induced maximum metabolic rate was significantly lower for 8 °C-acclimated cod when compared to both 12 °C-acclimated cod (*P* = 0.010) and haddock (*P* < 0.001), whereas the MMR_*T*_ of 12 °C-acclimated cod and haddock did not differ significantly (*P* = 0.165) (Table 1). Although neither RMR nor MMR_*T*_ of the 12 °C-acclimated cod and haddock differed significantly, there was a significant difference in AS_*T*_ between the two groups (*P* = 0.028), with haddock having a 36.3% larger AS_*T*_ (Table 1). In contrast, AS_*T*_ did not differ significantly between 8- and 12 °C-acclimated cod (*P* = 0.460) or between 8 °C-acclimated cod and 12 °C-acclimated haddock (*P* = 0.216).

**Figure 2:**
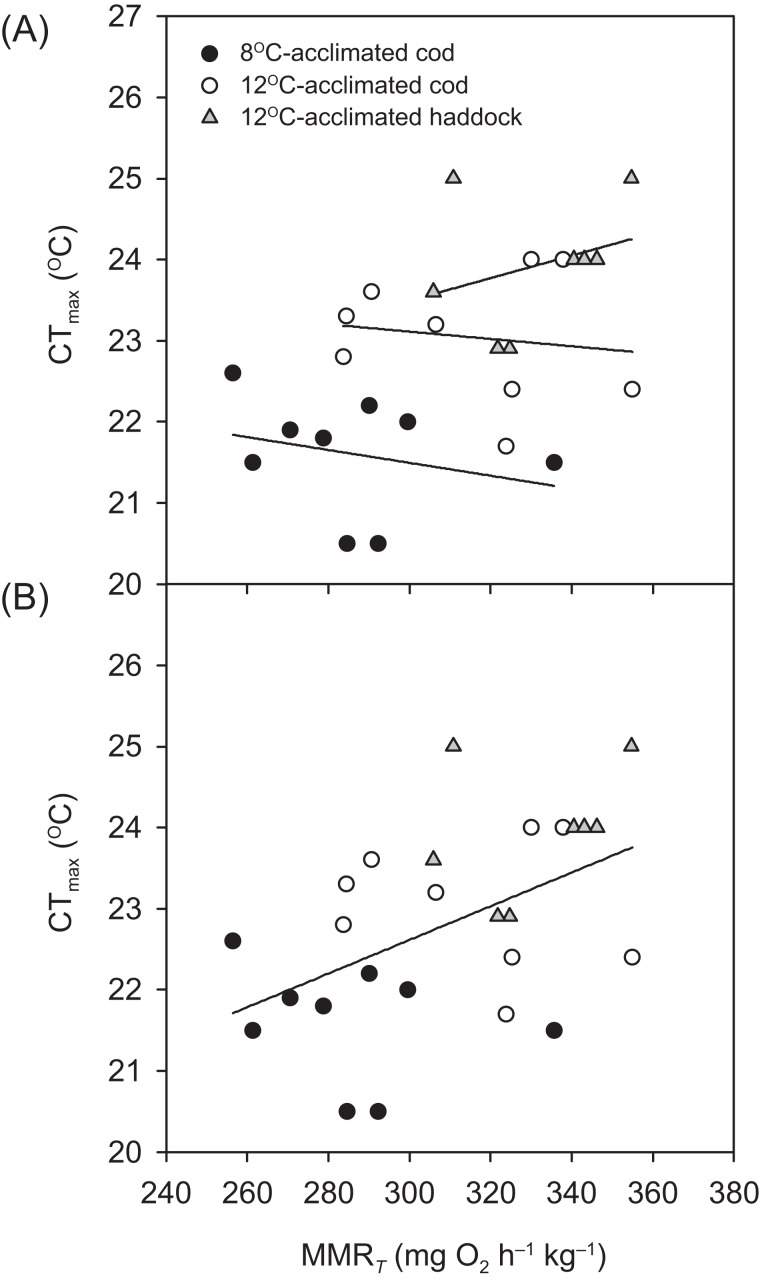
Temperature tolerance of cod and haddock in relation to their maximum metabolic rates. Critical thermal maxima (CT_max_) vs. temperature-induced maximum metabolic rates (MMR*_T_*) for individual cod acclimated to 8 °C (black circles; *n* = 9) or 12 °C (white circles; *n* = 9), and haddock acclimated to 12 °C (grey triangles; *n* = 8), as measured during the 2 °C h^−1^ warming challenges (i.e. CT_max_ tests). (A) There were no significant relationships between MMR*_T_* and CT_max_ for individual groups (*r*^2^ < 0.093, *P* > 0.463). However, (B) a high MMR*_T_* was associated with a high CT_max_ across groups and species (*r*^2^ = 0.244, *P* = 0.010), but CT_max_ was not related to either aerobic scope (*r*^2^ = 0.050, *P* = 0.270) or routine metabolic rate (*r*^2^ = 0.011, *P* = 0.610).

Critical thermal maximum was 1.6 °C lower for 8- than 12 °C-acclimated cod (*P* < 0.001) (i.e. 21.4 ± 0.3 vs. 23.0 ± 0.2 °C, respectively) (Table 1), indicating that acclimation temperature had a significant effect on the thermal tolerance of this species. CT_max_ was also significantly lower for 12 °C-acclimated cod than haddock (*P* = 0.031) (Table 1), but only by 0.9 °C on average, with values for individual cod ranging from 21.7 to 24.0 °C and those for individual haddock ranging from 22.9 to 25.0 °C. No significant relationships were observed between any of the metabolic rates (RMR, MMR_*T*_, or AS_*T*_) and CT_max_ within a particular group (linear regressions: *P* = 0.148–0.816 for RMR, 0.463–0.710 for MMR_*T*_, and 0.234–0.624 for AS_*T*_). However, MMR_*T*_ (but not RMR or AS_*T*_) was positively related to CT_max_ across groups and species (*r*^2^ = 0.244, *P* = 0.010) (Fig. 2).

### Metabolic rate and swimming performance

Both the SMR and the RMR for the three groups were significantly different (Table 2). The SMR of 12 °C-acclimated cod at 12 °C was higher than the SMR of 12 °C-acclimated haddock at 12 °C by 85.9% (*P* = 0.003), and the SMR of 12 °C-acclimated cod at 16 °C was higher than the SMR of both 12 °C-acclimated cod (*P* = 0.003) and haddock (*P* < 0.001) at 12 °C by 45.2% and 170.0%, respectively. The RMR of 12 °C-acclimated cod at 12 °C was higher than the RMR of 12 °C-acclimated haddock at 12 °C by 54.9% (*P* = 0.005), and the RMR of 12 °C-acclimated cod at 16 °C was higher than the RMR of both 12 °C-acclimated cod (*P* = 0.002) and haddock (*P* < 0.001) at 12 °C by 38.5% and 114.7%, respectively.

**Table 2:** Metabolic rate and swimming performance of cod and haddock. Standard metabolic rate (SMR), routine metabolic rate (RMR), swimming-induced maximum metabolic rate (MMR*_S_*), swimming-induced aerobic scope (AS*_S_*), critical swimming speed (*U*_crit_), optimal swimming speed (*U*_opt_), and minimum cost of transport (COT_min_) for 12 °C-acclimated cod and haddock swimming at 12 °C and 12 °C-acclimated cod swimming at 16 °C. Note that SMR was estimated by extrapolating the ${\rm }{\dot M_{{{\rm O}_2}}}$ vs. swimming speed relationship back to a swimming speed of zero. Metabolic rates scaled to a body mass of 100 g (using a metabolic mass-scaling coefficient of 0.80) are presented in italics in parentheses. Comparisons between groups were made using one-way ANOVAs followed by Holm–Sidak post hoc tests.

	Cod, 12 °C	Cod, 16 °C	Haddock, 12 °C
*n*	8	9	8
M (g)	67.6 ± 5.4^a^	61.3 ± 2.9^a^	63.0 ± 1.8^a^
L (cm)	20.9 ± 0.6^a^	20.9 ± 0.3^a^	18.7 ± 0.2^b^
K	0.73 ± 0.04^a^	0.67 ± 0.02^a^	0.96 ± 0.02^b^
SMR (mg O_2_ h^−1^ kg^−1^)	156.7 ± 15.2^b^ (*144.0 ± 13.8*^*b*^)	227.6 ± 19.4^a^ (*205.2 ± 16.0*^*a*^)	84.3 ± 4.2^c^ (*76.9 ± 4.1*^*c*^)
RMR (mg O_2_ h^−1^ kg^−1^)	185.0 ± 15.5^b^ (*170.0 ± 14.1*^*b*^)	256.3 ± 17.8^a^ (*231.1 ± 14.4*^*a*^)	119.4 ± 7.1^c^ (*108.9 ± 6.8*^*c*^)
MMR_*S*_ (mg O_2_ h^−1^ kg^−1^)	315.0 ± 13.8^a,b,(e)^ (*289.1 ± 9.8*^*a*^)	353.3 ± 16.2^a,(d)^ (*319.7 ± 14.5*^*a*^)	284.9 ± 6.0^b,(f)^ (*259.5 ± 4.7*^*b*^)
AS_*S*_ (mg O_2_ h^−1^ kg^−1^)	130.0 ± 18.6^a,b^ (*119.0 ± 16.6*^*a,b*^)	97.0 ± 18.0^a^ (*88.5 ± 16.8*^*a*^)	165.5 ± 12.3^b^ (*150.6 ± 11.0*^*b*^)
*U*_crit_ (cm s^−1^)	54.7 ± 1.4^a,(d)^	50.6 ± 1.9^a,(e)^	56.5 ± 1.4^a,(d)^
*U*_crit_ (BL s^−1^)	2.62 ± 0.09^a^	2.42 ± 0.08^a^	3.02 ± 0.09^b^
*U*_opt_ (cm s^−1^)	25.1 ± 2.3^a^	23.1 ± 1.4^a^	21.4 ± 0.7^a^
*U*_opt_ (BL s^−1^)	1.21 ± 0.12^a^	1.11 ± 0.06^a^	1.14 ± 0.04^a^
COT_min_ (mg O_2_ km^−1^ kg^−1^)	55.4 ± 7.7^a^	40.3 ± 10.9^a^	52.3 ± 7.0^a^

**Notes:**

a,b,c Significant differences between groups at *P* < 0.05

d,e,f Differences at *P* < 0.10 (pairwise comparisons).

*n*, number of experimental animals; M, body mass; L, fork length; K, condition factor. Values are means ± s.e.m.

**Figure 3:**
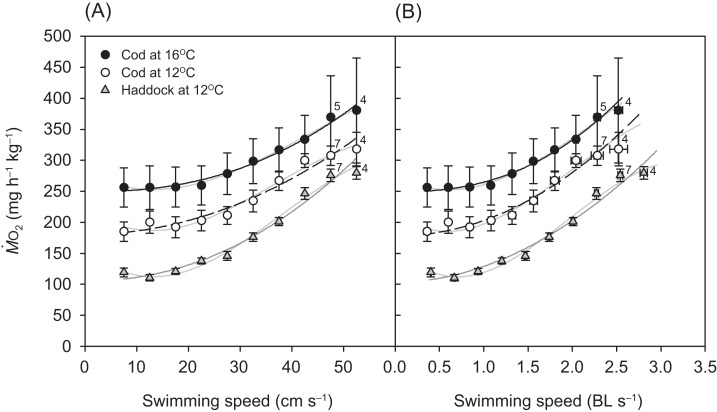
Metabolic rate of cod and haddock during swimming. Metabolic rate (${\rm }{\dot M_{{{\rm O}_2}}}$; means ± s.e.m.) vs. swimming speed (*U*) in either (A) cm per second (cm s^−1^) or (B) body lengths per second (BL s^−1^) for cod swimming at 12 °C (white circles, broken black line; *n* = 8), haddock swimming at 12 °C (grey triangles, solid grey line; *n* = 8), and cod swimming at 16 °C after an acute increase from 12 °C (black circles, solid black line; *n* = 9), as measured during the critical swimming speed (*U*_crit_) tests. Numbers next to symbols indicate reduced sample sizes due to some individuals fatiguing earlier than others at the highest swimming speeds. Although third-order polynomials (solid light grey lines in the background) fitted the data better, three-parameter power functions were used to describe the relationship between ${\rm }{\dot M_{{{\rm O}_2}}}$ and swimming speed as this is the hydro-dynamically most appropriate mathematical form of the relationship (cf., Papadopoulos, 2009; Horodysky et al., 2011; Roche et al., 2013). Equations in (A) are ${\rm }{\dot M_{{{\rm O}_2}}}$ = 180.47 + 0.055*U*^2.01^ (*r*^2^ = 0.530), ${\rm }{\dot M_{{{\rm O}_2}}}$ = 105.22 + 0.047*U*^2.11^ (*r*^2^ = 0.887), and ${\rm }{\dot M_{{{\rm O}_2}}}$ = 249.39 + 0.013*U*^2.35^ (*r*^2^ = 0.389) for cod swimming at 12 °C, haddock swimming at 12 °C, and cod swimming at 16 °C, respectively. Corresponding equations in (B) are ${\rm }{\dot M_{{{\rm O}_2}}}$ = 178.13 + 24.88*U*^2.04^ (*r*^2^ = 0.614), ${\rm }{\dot M_{{{\rm O}_2}}}$ = 102.98 + 25.98*U*^1.94^ (*r*^2^ = 0.863), and ${\rm }{\dot M_{{{\rm O}_2}}}$ = 249.18 + 15.64*U*^2.42^ (*r*^2^ = 0.398), respectively.

**Figure 4:**
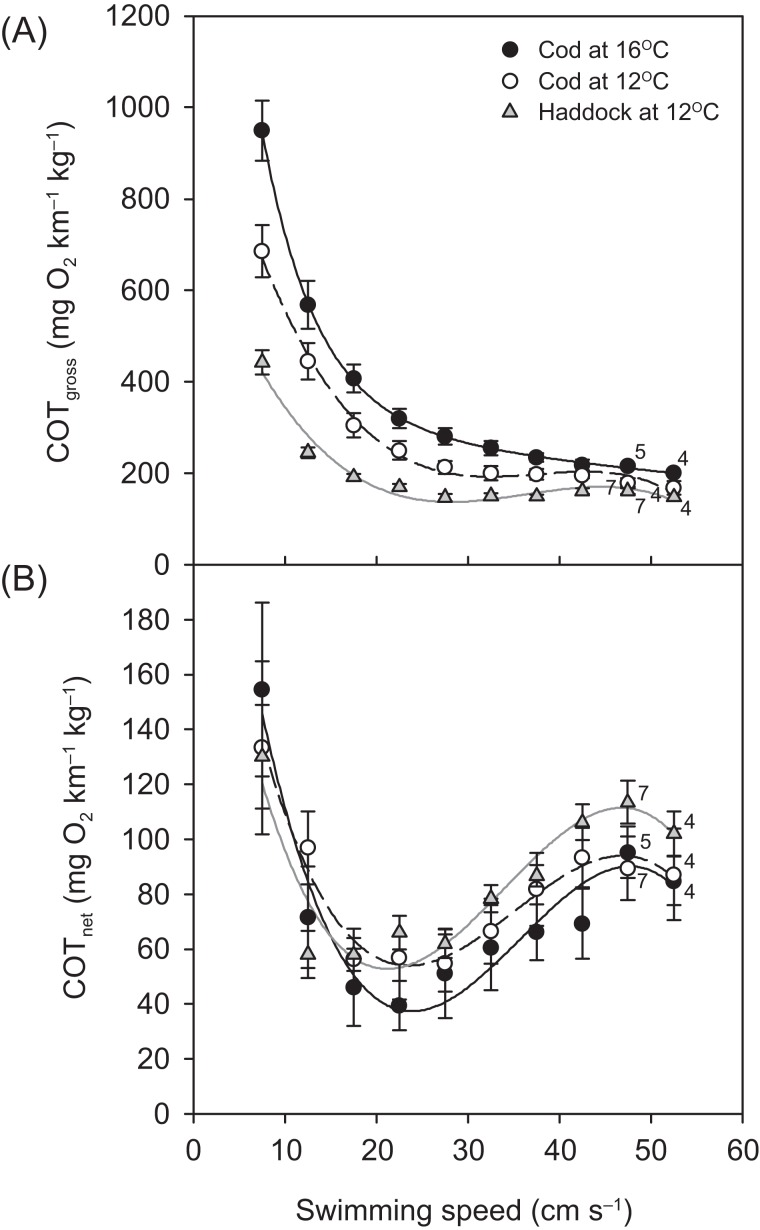
Cost of transport for cod and haddock at different swimming speeds. Gross (A) and net (B) cost of transport (COT, means ± s.e.m.) for cod swimming at 12 °C (white circles, broken black line; *n* = 8), haddock swimming at 12 °C (grey triangles, solid grey line; *n* = 8), and cod swimming at 16 °C after an acute increase from 12 °C (black circles, solid black line; *n* = 9), as measured during the critical swimming speed (*U*_crit_) tests. Numbers next to symbols indicate reduced sample sizes due to some individuals fatiguing earlier than others at the highest swimming speeds. Equations in (A) are: COT_gross_ = −0.0199*U*^3^ + 2.2186*U*^2^ – 80.926*U* + 1163.9 (*r*^2^ = 0.826), COT_gross_ = −0.0152*U*^3^ + 1.6427*U*^2^ – 56.22*U* + 757.76 (*r*^2^ = 0.875), and COT_gross_ = −0.0275*U*^3^ + 3.0753*U*^2^ – 112.87*U* + 1602.8 (*r*^2^ = 0.850) for cod swimming at 12 °C, haddock swimming at 12 °C, and cod swimming at 16 °C, respectively. Corresponding equations in (B) are: COT_net_ = −0.0064*U*^3^ + 0.6728*U*^2^ – 21.011*U* + 259.06 (*r*^2^ = 0.256), COT_net_ = −0.0070*U*^3^ + 0.7105*U*^2^ – 20.713*U* + 239.02 (*r*^2^ = 0.483), and COT_net_ = −0.0077*U*^3^ + 0.8259*U*^2^ – 26.197*U* + 297.88 (*r*^2^ = 0.313), respectively.

For all groups, the relationship between ${\dot M_{{{\rm O}_2}}}$ and swimming speed could be described by a hydrodynamic-based power function, although after 42.5 cm s^−1^, a slight plateauing of ${\dot M_{{{\rm O}_2}}}$ was observed prior to the fish reaching their *U*_crit_ (Fig. 3). Throughout the *U*_crit_ test, the ${\dot M_{{{\rm O}_2}}}$ of the three groups was in the order: cod at 16 °C > cod at 12 °C > haddock at 12 °C, and this pattern was reflected in the fish’s COT_gross_ (Fig. 4A). For example, COT_gross_ was 10.4–44.9% lower for haddock than for cod at 12 °C across swimming speeds.

Swimming-induced maximum metabolic rate (MMR_*S*_) followed the same general pattern as for RMR and tended to increase from 12 to 16 °C for cod (by 12.2%; *P* = 0.050) (Table 2). When comparing between species, the MMR_*S*_ of 12 °C-acclimated cod at 16 °C was significantly higher than the MMR_*S*_ of 12 °C-acclimated haddock at 12 °C (*P* = 0.001), while the MMR_*S*_ of both cod and haddock at 12 °C were the same (*P* = 0.127). AS_*S*_ tended to show a reverse pattern, with the AS_*S*_ of 12 °C-acclimated cod at 16 °C being significantly lower than the AS_*S*_ of 12 °C-acclimated haddock at 12 °C (*P* = 0.008), while the other groups did not differ significantly (Table 2). The same statistical differences in AS_*S*_ between groups were seen when using AS_*S*_ calculated as MMR_*S*_–SMR (results not presented), rather than MMR_*S*_–RMR, with AS_*S*_ being, on average, 17.5%, 17.9%, and 22.8% lower for 12 °C-acclimated haddock at 12 °C, 12 °C-acclimated cod at 12 °C, and 12 °C-acclimated cod at 16 °C, respectively, when calculated using RMR instead of SMR.

Although *U*_crit_ was slightly higher for cod at 12 °C when compared to cod at 16 °C, these values were not significantly different (*P* = 0.114) (Table 2). However, the haddock’s *U*_crit_ was significantly higher than that for cod at either 12 or 16 °C, but only when expressed as relative (BL s^−1^) swimming speed. This statistical discrepancy between relative vs. absolute *U*_crit_ was not surprising since the haddock were slightly shorter than the cod (Table 2).

Although COT_gross_ was consistently lower for the haddock as compared to the cod at 12 °C, and especially when compared to cod at 16 °C (Fig. 4A), COT_net_ (Fig. 4B) tended to show the opposite pattern. However, neither the *U*_opt_ nor the COT_min_ differed significantly between the three groups (*P* > 0.284) (Table 2).

## Discussion

The main goals of this study were to: (1) assess the effect of acclimation temperature (8 vs. 12 °C) on Atlantic cod thermal tolerance (CT_max_); (2) determine the effect of an acute increase in temperature (from 12 to 16 °C) on cod swimming and metabolic performance; and (3) compare all of these metrics between cod and haddock acclimated to the same temperature (12 °C).

As expected, the temperature to which the cod were exposed to prior to experiments had an important influence on their thermal tolerance; acclimation to a 4 °C higher temperature shifted the upper thermal limits upwards so that the mean CT_max_ observed for cod acclimated to 12 °C was 23.0 °C, which was 1.6 °C higher than for cod acclimated to 8 °C (CT_max_ = 21.4 °C) (Table 1). Bøhle (1974) found similar results for cod, with the lethal upper thermal limit for 50% of the fish (LT_50_) increasing from 19.5–20.0 °C for cod acclimated to 9 °C to 20.5 °C for cod acclimated to 16 °C. In addition, the 9 °C-acclimated cod had a mean mortality rate of approximately 5.8% min^−1^ when kept at 21 °C vs. a mortality rate of approximately 0.1% min^−1^ for the 16 °C-acclimated cod when exposed to the same temperature (Bøhle, 1974), emphasising that thermal history has an important effect on survival at extreme temperatures. The cod CT_max_ values from the present study also correspond well with those from Zanuzzo et al. (2019), where CT_max_ was 22.5 °C for 10 °C-acclimated cod experiencing the same rate of warming (2 °C h^−1^). Beitinger & Bennett (2000) compared 21 species of temperate fish and found that lethal temperatures increased with an increase in acclimation temperature at a rate of 0.5 °C per 1 °C increase in acclimation temperature. Most of the data from that study were based on freshwater species, which often show a larger window of thermal tolerance as compared to marine species. However, particularly in Northern hemisphere species such as the Atlantic cod, there is an ability to acclimatise and shift upper tolerance thresholds between seasons and in a latitudinal cline (Pörtner, 2002), and the CT_max_ results from the present study are in line with the thermal polygon proposed by Beitinger & Bennett (2000); i.e. a 4 °C difference in the cod’s acclimation temperature resulted in a 1.6 °C difference in CT_max_.

When evaluating differences between cod and haddock acclimated to the same temperature (12 °C), the results show that haddock are slightly (yet significantly) more tolerant of high temperatures; they had a mean CT_max_ that was 0.9 °C higher than for cod (23.9 vs. 23.0 °C, respectively). As critical temperatures differ between species depending on latitude or seasonal temperature acclimatisation, and are therefore related to geographical distribution (Wang & Overgaard, 2007; Beitinger, Bennett & McCauley, 2000; Pörtner, 2001), these results were somewhat expected. The upper limit of the temperature range at which haddock are usually found is 13 °C, and this is 2 °C higher than the upper temperature determining cod distribution (~11 °C; Brander, 1995; Dutil & Brander, 2003). The slight difference in heat tolerance is also supported by the effect of temperature on metabolic rate, as cod tended to show a plateau in oxygen uptake rate $({\dot M_{{{\rm O}_2}\hskip-1pt}})$ around 20 °C, whereas haddock did not show signs of impaired function until 22 °C (Fig. 1). These results suggest that haddock may be able to tolerate a 1–2 °C higher acute increase in temperature, as compared to cod, before experiencing significant thermal stress.

The concept of ‘oxygen- and capacity-limited thermal tolerance’ proposes that AS plays a key role in setting the thermal performance of ectothermic animals such as fishes, with performance (and resulting fitness) optimised at the temperature where AS is highest (Farrell, 2016; Pörtner & Farrell, 2008; Pörtner, Bock & Mark, 2017; Pörtner, 2010; Eliason et al., 2011), although concern has been expressed about the universality of this concept (summarised in Jutfelt et al. (2018)). Along the same principles, upper thermal tolerance has been proposed to be constrained by a failure of the cardiorespiratory system to support increases in maximum oxygen uptake (i.e. MMR) at high temperatures, thereby reducing AS (Pörtner & Farrell, 2008; Eliason et al., 2011; Farrell et al., 2009). Based on the observed thermal distribution of wild cod (Dutil & Brander, 2003)—as well as bioenergetic models that take into account metabolic rate, life history, and behaviour (Holt & Jørgensen, 2015)—the temperature where performance and fitness is optimised for adult Atlantic cod is believed to be around 10 °C. The present study shows that cod swimming at 16 °C (i.e. well above that ‘optimal’ temperature), after an acute increase in temperature from 12 °C, tended to have a lower AS_*S*_ than cod swimming at 12 °C (i.e. closer to the optimal temperature). However, the difference in AS_*S*_ between the two groups was not significant (*P* = 0.174) and the lower AS_*S*_ of cod at 16 °C was caused by a proportionally larger increase in RMR, rather than a decrease in MMR_*S*_. In fact, the MMR_*S*_ of cod swimming at 16 °C tended to be higher than the MMR_*S*_ of cod swimming at 12 °C (*P* = 0.050; Table 2). A more pronounced increase in the lower (i.e. resting or RMR) compared to the higher (i.e. MMR) end of the metabolic scale has previously been reported for cod acutely exposed to a 4 °C temperature increase (from 7 to 11 °C; Sylvestre et al., 2007), and is also seen in other fish species exposed to warming (Lefevre, 2016; Sandblom et al., 2016; Clark et al., 2011). The cod MMR_*S*_ values in the present study (315.0–353.3 mg O_2_ h^−1^ kg^−1^ at 12 to 16 °C) are comparable to those reported by Sylvestre et al. (2007) at 11 °C (~338 mg O_2_ h^−1^ kg^−1^ when adjusted for body mass) and are similar to long-term (several months) acclimated cod where MMR_*S*_ was lowest at 5 °C, higher at 10 °C, and highest (albeit also not significantly) at 15 °C (MMR_*S*_ values from ~303.6–319.6 mg O_2_ h^−1^ kg^−1^ at 10–15 °C, after adjusting for body mass and temperature; Schurmann & Steffensen, 1997). Taken together, these results suggest that thermal performance of cod at supra-optimal, but sub-lethal, temperatures is not limited by maximum oxygen transport capacity. This conclusion is supported by the lack of a relationship between CT_max_ and MMR_*T*_ within species and acclimation groups in the present study (Fig. 2A). Nonetheless, there was a positive relationship between CT_max_ and MMR_*T*_ across all groups and species (Fig. 2B), and this does suggest that thermal tolerance and the capacity for maximum oxygen uptake are related. This overall relationship between thermal tolerance and MMR_*T*_, combined with similar trends between the three groups of fish in terms of the plateauing (or slight decrease) of ${\dot M_{{{\rm O}_2}}}$ as temperatures approach CT_max_ (Fig. 1), suggests that the physiological processes that determine the critical temperature could be the same. In a study by Pörtner et al. (2001), heat stress in cod was shown to elicit a temperature-dependent decrease in venous, but not arterial, oxygen tensions $({P_{{{\rm O}_2}\hskip-1pt}})$, which suggests that, in these fish, the capacity for oxygen uptake at the gills may be maximised such that arterial oxygen uptake does not become limiting. However, the drop in venous ${P_{{{\rm O}_2}}}$ indicates that increased oxygen uptake from the blood during warming is not fully compensated for by circulatory performance (Pörtner et al., 2001). Sartoris et al. (2003) concluded that circulatory, rather than ventilatory, performance sets the limit of thermal tolerance in cod. Circulatory performance may thus become a limiting factor due to the temperature-dependent decrease in ${P_{{{\rm O}_2}}}$ in venous blood and its impact on cardiac performance (Farrell et al., 2009). In addition, haemoglobin oxygen affinity is reduced in cod as temperature increases, and it has been shown that at 20 °C the in vitro oxygen binding capacity of haemoglobin of cod acclimated to 7 °C was no longer enough to achieve maximum saturation, suggesting that decreased blood oxygen carrying capacity influenced thermal limits (Gollock et al., 2006). This relationship is supported by our finding that ${\dot M_{{{\rm O}_2}}}$ in the present study did not rise after 20 °C in cod acclimated to 12 °C (Fig. 1). Despite such potential cardio-respiratory oxygen limitations at extreme temperatures, the maintained capacity for maximum oxygen uptake of cod swimming at 16 vs. 12 °C (Table 2; Fig. 3) suggests that oxygen does not become the limiting factor for this species until close to lethal limits, which agrees with recent findings on both cold- and warm-water fishes (Holt & Jørgensen, 2015; Gräns et al., 2014; Norin, Malte & Clark, 2014; Lefevre, 2016). Finally, it is equally possible that the overall relationship between CT_max_ and MMR_*T*_ is driven by a loss of nervous function at extreme temperatures, rather than a causal oxygen limitation (Ern et al., 2015; Jutfelt et al., 2019). Such an effect could impair muscle function and reduce tissue oxygen demand, and thus explain the plateauing (or slight decreasing) of ${\dot M_{{{\rm O}_2}}}$ as temperatures approach CT_max_ (Fig. 1).

When comparing the CT_max_ and swimming experiments from the present study it is evident that 12 °C-acclimated cod reached the same MMR, and had the same AS, regardless of the method employed (i.e. temperature- and swimming-induced MMR were the same; Tables 1 and 2). These data support the findings of previous studies on Atlantic cod (e.g. Gollock et al., 2006 vs. Petersen & Gamperl, 2010, and Powell & Gamperl, 2016 show that AS_*T*_ is within 10–20% of AS_*S*_), and suggest that the physiological mechanisms responsible for increasing oxygen uptake to meet rising demands are similar during warming and physical activity in this species. The 12 °C-acclimated haddock, on the other hand, had a significantly lower RMR and MMR in the swimming experiment compared to the CT_max_ experiment, but maintained the same AS. The reason for these differences in RMR and MMR between the two haddock experiments, but not the cod experiments, is not known. However, they could potentially be related to the pre-experiment exercise protocol where the fish were ‘trained’ to swim against a constant current (~1 BL s^−1^) in their holding tank prior to introduction to the experimental swim flume respirometer. Since the cod is believed to be the inferior swimmer of the two species (see below) it is possible that exercise training improved the swimming efficiency of haddock (thereby lowering their ${\dot M_{{{\rm O}_2}}}$ at a constant swimming speed), but not of cod trained at ~1 BL s^−1^. Such an influence of training on ${\dot M_{{{\rm O}_2}}}$ may be related to muscle fibre dynamics, lowered levels of stress hormones, lowered energetically costly behavioural interactions (e.g. lowered aggressiveness) caused by schooling, as well as energetic savings from RAM ventilation (Farrell & Steffensen, 1987; Davidson, 1997, and references within). Regardless of the exact reasons behind the observed effect, both the RMR (119.4 ± 7.1 mg O_2_ h^−1^ kg^−1^) and MMR_*S*_ (284.9 ± 6.0 mg O_2_ h^−1^ kg^−1^) of the haddock from the swimming experiment are comparable to earlier studies on this species: Perez-Casanova, Lall & Gamperl (2010) report values for RMR of 103.6–107.0 mg O_2_ h^−1^ kg^−1^ for ~40 g haddock at 11 °C, and Tytler (1969) report MMR_*S*_ values at 10 °C of 276 ± 14 mg O_2_ h^−1^ kg^−1^ for ~156 g haddock.

The observed differences in metabolic rate between cod swimming at 12 and 16 °C were not directly reflected in their *U*_crit_, as *U*_crit_ did not differ significantly between fish at 12 °C (2.62 ± 0.08 BL s^−1^) and 16 °C (2.42 ± 0.08 BL s^−1^). The lack of observed differences in *U*_crit_ when cod are exposed to moderate to relatively high temperatures is in agreement with earlier studies on this species; although Schurmann & Steffensen (1997) and Sylvestre et al. (2007) used larger fishes (~30–48 cm), the relative *U*_crit_ recorded in those studies showed the same pattern with temperature (1.7 vs. 1.9 BL s^−1^ at 10 vs. 15 °C in Schurmann & Steffensen (1997) and ~1.6 BL s^−1^ at both 7 and 11 °C in Sylvestre et al. (2007)). It should be noted, however, that the apparent thermal insensitivity of *U*_crit_ over this temperature range (i.e. 7–15 °C) does not extend to lower temperatures, as both 5 °C-acclimated cod swimming at 5 °C (Schurmann & Steffensen, 1997) and 7 °C-acclimated cod swimming at 3 °C (Sylvestre et al., 2007) had significantly lower *U*_crit_ values than reported at the above-mentioned warmer temperatures in those studies. Although *U*_crit_ and maximum oxygen transport capacity (MMR_*S*_) in the present experiment did not differ significantly between the 12 °C-acclimated cod at 12 °C and those acutely exposed to 16 °C, the cod at 16 °C did show signs of struggling at the highest swimming speeds. This is evident from the greater variability in the data and the earlier reduction in the number of fish that were capable of swimming at speeds of 47.5 cm s^−1^ (2.28 BL s^−1^) and 52.5 cm s^−1^ (2.52 BL s^−1^) when compared to cod swimming at 12 °C (Fig. 3). That 16 °C is stressful for cod is also supported by studies which found increased plasma cortisol at this temperature (compared to 14 °C and lower) during both acute (Perez-Casanova et al., 2008a) and chronic (Perez-Casanova et al., 2008b) warming.

When observing the fish in the swim-tunnel, it appeared that the haddock was the superior swimmer of the two species, and this is reflected by the data. The haddock swimming at 12 °C had a higher *U*_crit_ than cod swimming at either 12 or 16 °C (Table 2; Fig. 3), and the swimming speed vs. metabolic rate relationships were clearly distinct between the two species (Fig. 3), as was the RMR (and MMR_*S*_ at *P* < 0.10). This conclusion is consistent with previous studies where cod were reported as being reluctant to swim inside a flume respirometer (Soofiani & Priede, 1985), whereas haddock swim strongly and uniformly in the same kind of swim tunnel (Tytler, 1969). The swimming speeds obtained for haddock in the present study also compare well to the maximum sustainable swimming speeds reported in the literature. Breen et al. (2004) reported that ~18 cm haddock at 9.9 °C could swim up to ~50.3 cm s^−1^ (or 2.8 BL s^−1^; mean of fish 25, 27, and 31 in their Table 1), although their results were obtained using a large annular tank in which haddock were stimulated to swim using a moving light pattern that was meant to mimic the mesh of an approaching fishing net. Tytler (1969) swam haddock in a Blažka swim flume respirometer, similar to the respirometer used in the present study, and found *U*_crit_ to be 52.1 cm s^−1^ (2.1 BL s^−1^) for 24.8 cm haddock swimming at 10 °C. In addition, Tytler (1978) compared the swimming performance of haddock to that of cod at 10 °C and found that cod fatigued earlier, below 1.5 BL s^−1^ (compared to 2.1 BL s^−1^ for the haddock). These data all support the conclusion that haddock are better swimmers than Atlantic cod. The inherent differences in swimming performance between haddock and cod could, among other things, be related to differences in body morphology (haddock being closer to the optimal shape for reduced drag; i.e. closer to a fineness ratio of ~4.5) (Webb, 1974; Videler, 1993; Martinez et al., 2003), muscle biochemistry (Martinez et al., 2003; Kolok, 1992), and cardiac function (Gamperl & Farrell, 2004; Claireaux et al., 2005). Due to the pre-experiment training protocol employed, differences in swimming performance could also be related to differences in trainability between species (Davidson, 1997).

In conclusion, the data from the present study show that, for cod, an acute increase in temperature from 12 to 16 °C (i.e. beyond the species’ optimal temperature; Cheung, Watson & Pauly, 2013; Holt & Jørgensen, 2015), does not have a negative effect on maximum oxygen transport capacity, but signs of impaired swimming (struggling) become evident at 16 °C; albeit without a significant reduction in *U*_crit_. Since cod are reported to utilise habitats that are not only warmer than what is considered optimal (Neat & Righton, 2007; Righton et al., 2010), but also hypoxic (Neuenfeldt, Andersen & Hinrichsen, 2009), it is apparent that cod may be faced with physiological trade-offs on a daily basis. Compared to cod, haddock reach a significantly higher relative swimming speed (*U*_crit_) at a relatively lower MMR_*S*_ and can be considered a more efficient swimmer; something that is also evident from their overall lower COT_gross_. For the size class of fish used in the present experiment, this could indicate that undersized haddock may be better than cod at escaping fishing gear, as a positive relationship between swimming speed and escapability has been demonstrated (He, 1993).

## Supplemental Information

10.7717/peerj.7784/supp-1 Supplemental Information 1 Raw data: Metabolic rate of cod and haddock during warming and their critical thermal maxima.

10.7717/peerj.7784/supp-2 Supplemental Information 2 Raw data: Metabolic rate of cod and haddock during swimming and their critical swimming speeds.

## Additional Information and Declarations

### Data Availability

The following information was supplied regarding data availability: The raw data are available in the Supplemental Files.

## References

[ref-1] Audzinjoyte A, Barneche DR, Baudron AR, Belmaker J, Clark TD, Marshall CT, Morrongiello JR, Van Rijn I (2019). Is oxygen limitation in warming waters a valid mechanism to explain the decreased body size in aquatic ectotherms?. Global Ecology and Biogeography.

[ref-2] Baudron AR, Needle CL, Marshall CT (2011). Implications of a warming North Sea for the growth of haddock *Melanogrammus aeglefinus*. Journal of Fish Biology.

[ref-3] Beaugrand G, Brander KM, Lindley JA, Souissi S, Reid PC (2003). Plankton effect on cod recruitment in the North Sea. Nature.

[ref-4] Begg GA, Martinesdottir G (2002). Environmental and stock effects on spatial distribution and abundance of mature cod *Gadus morhua*. Marine Ecology Progress Series.

[ref-5] Beitinger TL, Bennett WA (2000). Quantification of the role of acclimation temperature in temperature tolerance of fishes. Environmental Biology of Fishes.

[ref-6] Beitinger TL, Bennett WA, McCauley RW (2000). Temperature tolerance of North American freshwater fishes exposed to dynamic changes in temperature. Environmental Biology of Fishes.

[ref-7] Bøhle B (1974). Temperaturpreferance hos torsk (*Gadus morhua* L.). Fisken og Havet.

[ref-8] Bradshaw WE, Holzapfel CM (2006). Evolutionary responses to rapid climate change. Science.

[ref-9] Brander KM (1995). The effect of temperature on growth of Atlantic cod (*Gadus morhua* L.). ICES Journal of Marine Science.

[ref-10] Brander KM (2005). Cod recruitment is strongly affected by climate when stock biomass is low. ICES Journal of Marine Science.

[ref-11] Breen M, Dyson J, O’Neill FG, Jones E, Haigh M (2004). Swimming endurance of haddock (*Melanogrammus aeglefinus* L.) at prolonged and sustained swimming speeds, and its role in their capture by towed fishing gears. ICES Journal of Marine Science.

[ref-12] Brett JR (1964). The respiratory metabolism and swimming performance of young sockeye salmon. Journal of the Fisheries Research Board of Canada.

[ref-13] Brett JR (1971). Energetic responses of salmon to temperature. A study of some thermal relations in the physiology and freshwater ecology of sockeye salmon (*Oncorhynchus nerka*). American Zoologist.

[ref-14] Cardinale M, Svedäng H, Bartolino V, Maiorano L, Casini M, Linderholm H (2012). Spatial and temporal depletion of haddock and Pollack during the last century in the Kattgat-Skagerrak. Journal of Applied Ichthyology.

[ref-15] Castonguay M, Rollet C, Fréchet A, Gagnon P, Gilbert D, Brêthes JC (1999). Distribution changes of Atlantic cod (*Gadus morhua*) in the Northern Gulf of St. Lawrence in relation to an oceanic cooling. ICES Journal of Marine Science.

[ref-16] Cavole LM, Demko AM, Diner RE, Giddings A, Koester I, Pagniello CMLS, Paulsen M-L, Ramirez-Valdez A, Schwenck SM, Yen NK, Zill ME, Franks PJS (2016). Biological impacts of the 2013–2015 warm-water anomaly in the Northeast Pacific: winners, losers, and the future. Oceanography.

[ref-17] Cheung WWL, Lam VWY, Sarmiento JL, Kearney K, Watson R, Pauly D (2009). Projecting global marine biodiversity impacts under climate change scenarios. Fish and Fisheries.

[ref-18] Cheung WWL, Sarmiento JL, Dunne J, Frölicher TL, Lam VWY, Palomares MLD, Watson R, Pauly D (2013). Shrinking of fishes exacerbates impacts of global ocean changes on marine ecosystems. Nature Climate Change.

[ref-19] Cheung WWL, Watson R, Pauly D (2013). Signature of ocean warming in global fisheries catch. Nature.

[ref-20] Claireaux G, McKenzie DJ, Genge AG, Chatelier A, Aubin J, Farrell AP (2005). Linking swimming performance, cardiac pumping ability and cardiac anatomy in rainbow trout. Journal of Experimental Biology.

[ref-21] Claireaux G, Webber DM, Kerr SR, Boutilier RG (1995). Physiology and behavior of free-swimming Atlantic cod (*Gadus morhua*) facing fluctuating temperature conditions. Journal of Experimental Biology.

[ref-22] Claireaux G, Webber DM, Lagardère J-P, Kerr SR (2000). Influence of water temperature and oxygenation on the aerobic metabolic scope of Atlantic cod (*Gadus morhua*). Journal of Sea Research.

[ref-23] Clark TD, Jeffries KM, Hinch SG, Farrell AP (2011). Exceptional aerobic scope and cardiovascular performance of pink salmon (*Oncorhynchus gorbuscha*) may underlie resilience in a warming climate. Journal of Experimental Biology.

[ref-24] Clark TD, Sandblom E, Jutfelt F (2013). Aerobic scope measurements of fishes in an era of climate change: respirometry, relevance and recommendations. Journal of Experimental Biology.

[ref-25] Daufresne M, Lengfellner K, Sommer U (2009). Global warming benefits the small in aquatic ecosystems. Proceedings of the National Academy of Sciences of the United States of America.

[ref-26] Davidson W (1997). The effects of exercise training on teleost fish, a review of recent literature. Comparative Biochemistry and Physiology Part A: Physiology.

[ref-27] Dutil J-D, Brander K (2003). Comparing productivity of North Atlantic cod (*Gadus morhua*) stocks and limits to growth production. Fisheries Oceanography.

[ref-28] Eliason EJ, Clark TD, Hague MJ, Hanson LM, Gallagher ZS, Jeffries KM, Gale MK, Patterson DA, Hinch SG, Farrell AP (2011). Differences in thermal tolerance among sockeye salmon populations. Science.

[ref-29] Eriksen E, Ingvaldsen R, Stiansen JE, Johansen GO (2012). Thermal habitat for 0-group fish in the Barents Sea; how climate variability impacts their density, length, and geographic distribution. ISEC Journal of Marine Science.

[ref-30] Ern R, Huong DTT, Phuong NT, Madsen PT, Wang T, Bayley M (2015). Some like it hot: thermal tolerance and oxygen supply capacity in two eurythermal crustaceans. Scientific Reports.

[ref-31] Farrell AP (2007). Cardiorespiratory performance during prolonged swimming tests with salmonids: a perspective on temperature effects and potential analytical pitfalls. Philosophical Transactions of the Royal Society B: Biological Sciences.

[ref-32] Farrell AP (2016). Pragmatic perspective on aerobic scope: peaking, plummeting, pejus and apportioning. Journal of Fish Biology.

[ref-33] Farrell AP, Eliason EJ, Sandblom E, Clark TD (2009). Fish cardiorespiratory physiology in an era of climate change. Canadian Journal of Zoology.

[ref-34] Farrell AP, Steffensen JF (1987). An analysis of the energetic cost of the branchial and cardiac pumps during sustained swimming in trout. Fish Physiology and Biochemistry.

[ref-35] Fossheim M, Primicerio R, Johannesen E, Ingvaldsen RB, Aschan MM, Dolgov AV (2015). Recent warming leads to a rapid borealization of fish communities in the Arctic. Nature Climate Change.

[ref-36] Fry FEJ, Hoar WS, Randall DJ (1971). The effects of environmental factors on the physiology of fish. Fish Physiology: Environmental Relations and Behaviour.

[ref-37] Gamperl AK, Farrell AP (2004). Cardiac plasticity in fishes: environmental influences and intraspecific differences. Journal of Experimental Biology.

[ref-38] Gamperl AK, Rodnick KJ, Faust HA, Venn EC, Bennett MT, Crawshaw LI, Keeley ER, Powell MS, Li HW (2002). Metabolism, swimming performance, and tissue biochemistry of high desert redband trout (*Oncorhynchus mykiss* ssp.): evidence for phenotypic differences in physiological function. Physiological and Biochemical Zoology.

[ref-39] Gollock MJ, Currie S, Petersen LH, Gamperl AK (2006). Cardiovascular and haematological responses of Atlantic cod (*Gadus morhua*) to acute temperature increase. Journal of Experimental Biology.

[ref-40] Gräns A, Jutfelt F, Sandblom E, Jönsson E, Wiklander K, Seth H, Olsson C, Dupont S, Ortega-Martinez O, Einarsdottir I, Björnsson BT, Sundell K, Axelsson M (2014). Aerobic scope fails to explain the detrimental effects on growth resulting from warming and elevated CO_2_ in Atlantic halibut. Journal of Experimental Biology.

[ref-41] He P (1993). Swimming speeds of marine fish in relation to fishing gears. ICES Marine Science Symposium.

[ref-42] Hofmann GE, Todgham AE (2010). Living in the now: physiological mechanisms to tolerate a rapidly changing environment. Annual Review of Physiology.

[ref-43] Hollins J, Thambithurai D, Koeck B, Crespel A, Bailey DM, Cooke SJ, Lindström J, Parsons KJ, Killen SS (2018). A physiological perspective on fisheries-induced evolution. Evolutionary Applications.

[ref-44] Holt RE, Jørgensen C (2015). Climate change in fish: effects of respiratory constraints on optimal life history and behavior. Biology Letters.

[ref-45] Horodysky AZ, Brill RW, Bushnell PG, Musick JA, Latour RJ (2011). Comparative metabolic rates of common western North Atlantic Ocean sciaenid fishes. Journal of Fish Biology.

[ref-46] Jutfelt F, Norin T, Ern R, Overgaard J, Wang T, McKenzie DJ, Lefevre S, Nilsson Göran E, Metcalfe NB, Hickey AJR, Brijs J, Speers-Roesch B, Roche DG, Gamperl AK, Raby GD, Morgan R, Esbaugh AJ, Gräns A, Axelsson M, Ekström A, Sandblom E, Binning SA, Hicks JW, Seebacher F, Jørgensen C, Killen SS, Schulte PM, Clark TD (2018). Oxygen- and capacity-limited thermal tolerance: blurring ecology and physiology. Journal of Experimental Biology.

[ref-103] Jutfelt F, Roche DG, Clark TD, Norin T, Binning SA, Speers-Roesch B, Amcoff M, Morgan R, Andreassen AH, Sundin J Brain cooling marginally increases acute upper thermal tolerance in Atlantic cod. Journal of Experimental Biology.

[ref-47] Kelly NI, Alzaid A, Nash GW, Gamperl AK (2014). Metabolic depression in cunner (*Tautogolabrus adspersus*) is influenced by ontogeny, and enhances thermal tolerance. PLOS ONE.

[ref-48] Killen SS (2014). Growth trajectory influences temperature preference in fish through an effect on metabolic rate. Journal of Animal Ecology.

[ref-49] Kobler A, Klefoth T, Mehner T, Arlinghaus R (2009). Coexistence of behavioural types in an aquatic top predator: a response to resource limitation?. Oecologia.

[ref-50] Kolok AS (1992). Morphological and physiological correlates with swimming performance in juvenile largemouth bass. American Journal of Physiology.

[ref-51] Lankin KF, Peck MA, Buckley LJ, Bengtson DA (2008). The effects of temperature, body size and growth rate on energy losses due to metabolism in early life stages of haddock (*Melanogrammus aeglefinus*). Marine Biology.

[ref-52] Lefevre S (2016). Are global warming and ocean acidification conspiring against marine ectotherms? A meta-analysis of the respiratory effects of elevated temperature, high CO_2_ and their interaction. Conservation Physiology.

[ref-53] Lima FP, Wethey DS (2012). Three decades of high-resolution coastal sea surface temperatures reveal more than warming. Nature Communications.

[ref-54] Malmberg SA, Blindheim J (1994). Climate, cod and capelin in northern waters. ICES Marine Science Symposia.

[ref-55] Martinez M, Guderley H, Dutil JD, Winger P, He P, Walsh SJ (2003). Condition, prolonged swimming performance and muscle metabolic capacities of cod (*Gadus morhua*). Journal of Experimental Biology.

[ref-56] Morita K, Fukuwaka M-A, Tanimata N, Yamamura O (2010). Size-dependent thermal preferences in a pelagic fish. Oikos.

[ref-57] Neat F, Righton D (2007). Warm water occupancy by North Sea cod. Proceedings of the Royal Society B: Biological Sciences.

[ref-58] Neuenfeldt S, Andersen KH, Hinrichsen H-H (2009). Some Atlantic cod *Gadus morhua* in the Baltic Sea visit hypoxic water briefly but often. Journal of Fish Biology.

[ref-59] Norin T, Malte H, Clark TD (2014). Aerobic scope does not predict the performance of a tropical eurythermal fish at elevated temperatures. Journal of Experimental Biology.

[ref-60] Ottersen G, Michaelsen K, Nakken O (1998). Ambient temperature and distribution of north-east Arctic cod. ICES Journal of Marine Science.

[ref-61] Papadopoulos A (2009). Hydrodynamics-based functional forms of activity metabolism: a case for the power-law polynomial function in animal swimming energetics. PLOS ONE.

[ref-62] Peck MA, Buckley LJ, Bengtson DA (2005). Effects of temperature, body size and feeding on rates of metabolism in young-of-the-year haddock. Journal of Fish Biology.

[ref-63] Pecl GT, Araújo MB, Bell JD, Blanchard J, Bonebrake TC, Chen I-C, Clark TD, Colwell RK, Danielsen F, Evengård B, Falconi L, Ferrier S, Frusher S, Garcia RA, Griffis RB, Hobday AJ, Janion-Scheepers C, Jarzyna MA, Jennings S, Lenoir J, Linnetved HI, Martin VY, McCormack PC, McDonald J, Mitchell NJ, Mustonen T, Pandolfi JM, Pettorelli N, Popova E, Robinson SA, Scheffers BR, Shaw JD, Sorte CJB, Strugnell JM, Sunday JM, Tuanmu M-N, Vergés A, Villanueva C, Wernberg T, Wapstra E, Williams SE (2017). Biodiversity redistribution under climate change: impacts on ecosystems and human well-being. Science.

[ref-64] Perez-Casanova JC, Afonso LOB, Johnson SC, Currie S, Gamperl AK (2008a). The stress and metabolic responses of juvenile Atlantic cod *Gadus morhua* L. to an acute thermal challenge. Journal of Fish Biology.

[ref-65] Perez-Casanova JC, Lall SP, Gamperl AK (2010). Effects of dietary protein and lipid level, and water temperature, on the post-feeding oxygen consumption of Atlantic cod and haddock. Aquaculture Research.

[ref-66] Perez-Casanova JC, Rise ML, Dixon B, Afonso LOB, Hall JR, Johnson SC, Gamperl AK (2008b). The immune and stress responses of Atlantic cod to long-term increases in water temperature. Fish & Shellfish Immunology.

[ref-67] Perry AL, Low PJ, Ellis JR, Reynolds JD (2005). Climate change and distribution shifts in marine fishes. Science.

[ref-68] Petersen LH, Gamperl AK (2010). Cod (*Gadus morhua*) cardiorespiratory physiology and hypoxia tolerance following acclimation to low-oxygen conditions. Physiological and Biochemical Zoology.

[ref-69] Pörtner HO (2001). Climate change and temperature-dependent biogeography: oxygen limitation of thermal tolerance in animals. Naturwissenschaften.

[ref-70] Pörtner HO (2002). Climate variations and the physiological basis of temperature dependent biogeography: systemic to molecular hierarchy of thermal tolerance in animals. Comparative Biochemistry and Physiology Part A: Molecular & Integrative Physiology.

[ref-71] Pörtner H-O (2010). Oxygen- and capacity-limitation of thermal tolerance: a matrix for integrating climate-related stressor effects in marine ecosystems. Journal of Experimental Biology.

[ref-72] Pörtner HO, Berdal B, Blust R, Brix O, Colosimo A, De Wachter B, Giuliani A, Johansen T, Fischer T, Knust R, Lannig G, Naevdal G, Nedenes A, Nyhammer G, Sartoris FJ, Serendero I, Sirabella P, Thorkildsen S, Zakhartsev M (2001). Climate induced temperature effects on growth performance, fecundity and recruitment in marine fish: developing a hypothesis for cause and effect relationships in Atlantic cod (*Gadus morhua*) and common eelpout (*Zoarces viviparus*). Continental Shelf Research.

[ref-73] Pörtner H-O, Bock C, Mark FC (2017). Oxygen- and capacity-limited thermal tolerance: bridging ecology and physiology. Journal of Experimental Biology.

[ref-74] Pörtner H-O, Bock C, Mark FC (2018). Connecting to ecology: a challenge for comparative physiologists? Response to ‘Oxygen- and capacity-limited thermal tolerance: blurring ecology and physiology’. Journal of Experimental Biology.

[ref-75] Pörtner HO, Farrell AP (2008). Physiology and climate change. Science.

[ref-76] Pörtner HO, Karl DM, Boyd PW, Cheung WWL, Lluch-Cota SE, Nojiri Y, Schmidt DN, Zavialov PO, Field CB, Barros VR, Dokken DJ, Mach KJ, Mastrandrea MD, Bilir TE, Chatterjee M, Ebi KL, Estrada YO, Genova RC, Girma B, Kissel ES, Levy AN, MacCracken S, Mastrandrea PR, White LL (2014). Ocean systems. Climate Change 2014: Impacts, Adaptation, and Vulnerability. Part A: Global and Sectoral Aspects. Contribution of Working Group II to the Fifth Assessment Report of the Intergovernmental Panel on Climate Change.

[ref-77] Pörtner HO, Knust R (2007). Climate change affects marine fishes through the oxygen limitation of thermal tolerance. Science.

[ref-78] Pörtner HO, Peck MA (2010). Climate change effects on fishes and fisheries: towards a cause-and-effect understanding. Journal of Fish Biology.

[ref-79] Powell MD, Gamperl AK (2016). Effects of *Loma morhua* (Microsporidia) infection on the cardiorespiratory performance of Atlantic cod *Gadus morhua* (L). Journal of Fish Diseases.

[ref-80] Reidy SP, Nelson JA, Tang Y, Kerr SR (1995). Post-exercise metabolic rate in Atlantic cod (*Gadus morhua*) and its dependence upon the method of exhaustion. Journal of Fish Biology.

[ref-81] Renaud PE, Berge J, Varpe Ø, Lønne OJ, Nahrgang J, Ottesen C, Hallanger I (2012). Is the poleward expansion by Atlantic cod and haddock threatening native polar cod, *Boreogadus saida*?. Polar Biology.

[ref-82] Righton DA, Andersen KH, Neat F, Thorsteinsson V, Steingrund P, Svedäng H, Michalsen K, Hinrichsen H-H, Bendall V, Neuenfeldt S, Wright P, Jonsson P, Huse G, Van Der Kooij J, Mosegaard H, Hüssy K, Metcalfe J (2010). Thermal niche of Atlantic cod *Gadus morhua*: limits, tolerance and optima. Marine Ecology Progress Series.

[ref-83] Rijn I, Buba Y, DeLong J, Kiflawi M, Belmaker J (2017). Large but uneven reduction in fish size across species in relation to changing sea temperatures. Global Change Biology.

[ref-84] Roche DG, Binning SA, Bosiger Y, Johansen JL, Rummer JL (2013). Finding the best estimates of metabolic rates in a coral reef fish. Journal of Experimental Biology.

[ref-85] Rogers LA, Stige LC, Olsen EM, Knutsen H, Chan K-S, Stenseth NC (2011). Climate and population density drive changes in body size throughout a century on the Norwegian coast. Proceedings of the National Academy of Sciences of the United States of America.

[ref-86] Sandblom E, Clark TD, Gräns A, Ekström A, Brijs J, Sundström LF, Odelström A, Adill A, Aho T, Jutfelt F (2016). Physiological constraints to climate warming in fish follow principles of plastic floors and concrete ceilings. Nature Communications.

[ref-87] Sartoris FJ, Bock C, Serendero I, Lannig G, Pörtner HO (2003). Temperature-dependent changes in energy metabolism, intracellular pH and blood oxygen tension in the Atlantic cod. Journal of Fish Biology.

[ref-88] Saunders RL (1963). Respiration of the Atlantic cod. Journal of the Fisheries Research Board of Canada.

[ref-89] Schulte PM (2015). The effects of temperature on aerobic metabolism: towards a mechanistic understanding of the responses of ectotherms to a changing environment. Journal of Experimental Biology.

[ref-90] Schurmann H, Steffensen JF (1997). Effects of temperature, hypoxia and activity on the metabolism of juvenile Atlantic cod. Journal of Fish Biology.

[ref-91] Soofiani NM, Priede IG (1985). Aerobic metabolic scope and swimming performance in juvenile cod, *Gadus morhua* L. Journal of Fish Biology.

[ref-92] Speers-Roesch B, Norin T (2016). Ecological significance of thermal tolerance and performance in fishes: new insights from integrating field and laboratory approaches. Functional Ecology.

[ref-93] Steffensen JF (1989). Some errors in respirometry of aquatic breathers: how to avoid and correct for them. Fish Physiology and Biochemistry.

[ref-94] Sylvestre E-L, Lapointe D, Dutil J-D, Guderley H (2007). Thermal sensitivity of metabolic rates and swimming performance in two latitudinally separated populations of cod, *Gadus morhua* L. Journal of Comparative Physiology B.

[ref-95] Thambithurai D, Hollins J, Van Leeuwen T, Rácz A, Lindström J, Parsons K, Killen SS (2018). Shoal size as a key determinant of vulnerability to capture under a simulated fishery scenario. Ecology and Evolution.

[ref-96] Tirsgaard B, Behrens JW, Steffensen JF (2015). The effect of temperature and body size on metabolic scope of activity in juvenile Atlantic cod *Gadus morhua* L. Comparative Biochemistry and Physiology Part A: Molecular & Integrative Physiology.

[ref-97] Tytler P (1969). Relationship between oxygen consumption and swimming speed in the haddock, *Melanogrammus aeglefinus*. Nature.

[ref-98] Tytler P, McLusky DS, Berry AJ (1978). The influence of swimming performance on the metabolic rate of gadoid fish. Physiology and Behavious of Marine Organisms.

[ref-99] Videler JJ (1993). Fish swimming.

[ref-100] Wang T, Overgaard J (2007). The heartbreak of adapting to global warming. Science.

[ref-101] Webb PW (1974). Hydrodynamics and energetics of fish propulsion. Bulletin of the Fisheries Research Board of Canada.

[ref-102] Zanuzzo FS, Bailey JA, Garber AF, Gamperl AK (2019). The acute and incremental thermal tolerance of Atlantic cod (*Gadus morhua*) families under normoxia and mild hypoxia. Comparative Biochemistry and Physiology Part A: Molecular & Integrative Physiology.

